# The Return on Investment of a Medicaid Tobacco Cessation Program in Massachusetts

**DOI:** 10.1371/journal.pone.0029665

**Published:** 2012-01-06

**Authors:** Patrick Richard, Kristina West, Leighton Ku

**Affiliations:** 1 Department of Health Policy, School of Public Health and Health Services, The George Washington University, Washington, District of Columbia, United States of America; 2 Center for Health Policy Research, School of Public Health and Health Services, The George Washington University, Washington, District of Columbia, United States of America; Finnish Institute of Occupational Health, Finland

## Abstract

**Background and Objective:**

A high proportion of low-income people insured by the Medicaid program smoke. Earlier research concerning a comprehensive tobacco cessation program implemented by the state of Massachusetts indicated that it was successful in reducing smoking prevalence and those who received tobacco cessation benefits had lower rates of in-patient admissions for cardiovascular conditions, including acute myocardial infarction, coronary atherosclerosis and non-specific chest pain. This study estimates the costs of the tobacco cessation benefit and the short-term Medicaid savings attributable to the aversion of inpatient hospitalization for cardiovascular conditions.

**Methods:**

A cost-benefit analysis approach was used to estimate the program's return on investment. Administrative data were used to compute annual cost per participant. Data from the 2002–2008 Medical Expenditure Panel Survey and from the Behavioral Risk Factor Surveillance Surveys were used to estimate the costs of hospital inpatient admissions by Medicaid smokers. These were combined with earlier estimates of the rate of reduction in cardiovascular hospital admissions attributable to the tobacco cessation program to calculate the return on investment.

**Findings:**

Administrative data indicated that program costs including pharmacotherapy, counseling and outreach costs about $183 per program participant (2010 $). We estimated inpatient savings per participant of $571 (range $549 to $583). Every $1 in program costs was associated with $3.12 (range $3.00 to $3.25) in medical savings, for a $2.12 (range $2.00 to $2.25) return on investment to the Medicaid program for every dollar spent.

**Conclusions:**

These results suggest that an investment in comprehensive tobacco cessation services may result in substantial savings for Medicaid programs. Further federal and state policy actions to promote and cover comprehensive tobacco cessation services in Medicaid may be a cost-effective approach to improve health outcomes for low-income populations.

## Introduction

Smoking is a leading cause of preventable death in the United States, resulting in an estimated 450,000 annual premature deaths, or nearly one of every five deaths. It is responsible for roughly 30% of all cancer deaths, for nearly 80% of deaths from chronic obstructive pulmonary disease, and for early cardiovascular disease deaths [1]–[3]. More than one-third of the smoking-attributable years of potential life lost are related to cardiovascular disease [4]. The annual economic burden of smoking in the U.S. has been estimated at nearly $193 billion in direct medical costs and productivity losses [2]. While the life-time prevalence rate for adult smokers in the U.S. population is about 20% of this rate is about twice as high among adults insured by Medicaid [1]–[3]. Smoking-related medical costs are responsible for 11% of Medicaid expenditures, representing an estimated $22 billion in 2004 [5].

Federal policy has sought to reduce smoking by Medicaid beneficiaries as an important public health goal. For instance, one of the key objectives of Healthy People 2020 is to “increase comprehensive Medicaid insurance coverage of evidence-based treatment for nicotine dependency in States and the District of Columbia [6].” Considerable efforts have been made at the state level to reduce smoking. In 2009, Medicaid programs in 47 states and the District of Columbia offered at least some form of coverage for tobacco-dependence treatments, although most had a limited range of benefits [7]. The Patient Protection and Affordable Care Act will increase this coverage; it requires all states to offer comprehensive tobacco cessation services for pregnant women as of 2010 (Section 4107 of the Act) and to cover anti-smoking medications under Medicaid by 2014 (Section 2502).

The state of Massachusetts initiated early efforts to provide comprehensive tobacco cessation medications and services to low-income Medicaid enrollees under its Tobacco Cessation & Prevention Program, starting in 2006. Under the program, with a physician's prescription, Medicaid beneficiaries could obtain FDA-approved smoking cessation medications with a copayment ranging from $1 to $3 per month. No preauthorization was required for a nicotine patch, gum or lozenge, bupropion (e.g., Zyban) or varenicline (Chantix) [8]. Massachusetts also offered up to five sessions of free telephone counseling for the state's quit line (although this was not required to get medications).

Research by Thomas Land, et al. found that this program reached a substantial share of smokers in Medicaid, achieving about a 37% use rate, and was successful in contributing to a 10% reduction in the rate of smoking by Medicaid beneficiaries [9]. Further analyses by Land, et al. examined the inpatient hospital utilization of Medicaid enrollees who used the smoking cessation benefit. The study used generalized estimating equations to examine changes in hospitalization trends among 21,656 Medicaid beneficiaries before and after the use of the tobacco cessation benefit, adjusting for demographics, comorbidities, seasonality, and other factors. On average, study participants were followed over four years, with 70 weeks in the post-benefit period. The study found that participation in the program was associated with statistically significant reductions of 46% in hospital inpatient admissions for acute myocardial infarction (AMI) (p<.05), 49% for coronary atherosclerosis and other heart disease (p<.05), and 32% for non-specific chest pain (p<.1), relative to the rate without the benefit [10]. There were no significant differences in hospitalizations for respiratory conditions or other seven other diagnostic groups evaluated.

In this study, we estimated the economic value of Massachusetts' tobacco cessation program's reduction on cardiovascular hospitalizations relative to program costs. We use the estimate of reductions in cardiovascular hospitalizations reported in Land's inpatient study [10]. Previous research has examined the efficacy of smoking cessation methods and found that pharmacotherapy can be a cost-effective treatment modality [11]–[18]. A recent study by Ladapo simulated the lifetime cost-effectiveness of a smoking counseling program for smokers hospitalized with AMI and concluded that counseling would reduce hospitalization costs but might increase lifetime healthcare costs by extending longevity [19]. In contrast, our study focuses on prevention of cardiovascular problems among smokers prior to hospitalization, primarily using pharmacotherapy, and focuses on short-term costs and savings, as opposed to lifetime cost-effectiveness. This study does not seek to measure all potential long-term savings due to the implementation of the tobacco cessation program, but represents a conservative estimate of short-term savings solely related to the avoidance of inpatient hospital admissions and treatment of cardiovascular diseases among Massachusetts Medicaid beneficiaries and smokers.

## Methods

### Objective

This study provides an independent estimate of cost savings and the return on investment (ROI) associated with reductions in inpatient hospital admissions for cardiovascular conditions by Medicaid beneficiaries participating in the Massachusetts Tobacco Cessation & Prevention Program from 2007 to 2009. It focuses on the costs and savings from the perspective of the Medicaid program.

### Study Design and Analytical Framework

**Figure 1:**
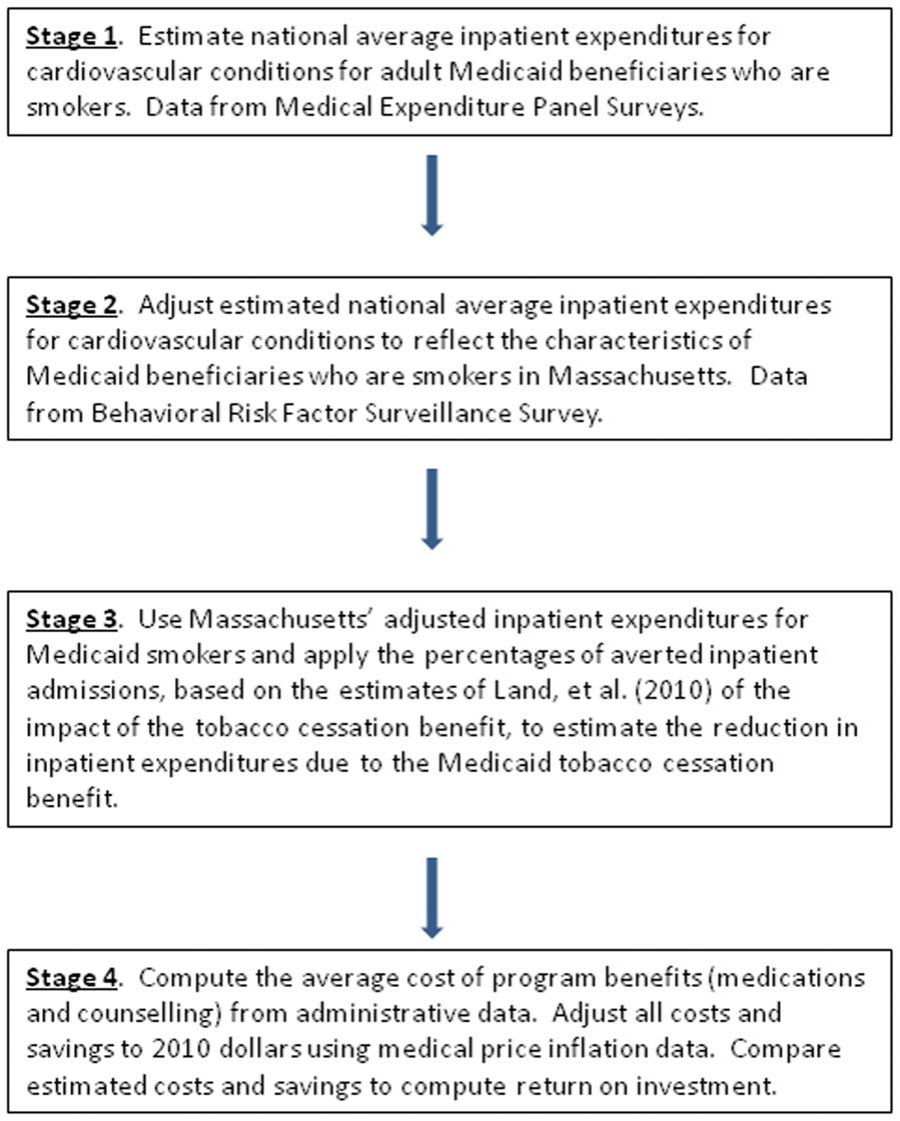
Flowchart summarizing the analyses.

This study uses cost-benefit analysis to estimate short-term ROI of the Massachusetts tobacco cessation benefit, based on estimated program costs and savings attributable to reduced cardiovascular admissions among adult Medicaid enrollees. We used a blend of national and state data to estimate costs and savings, as described in the data section below. National data sources include the Medical Expenditure Panel Survey (MEPS), while state data include administrative program cost data, the Massachusetts Behavioral Risk Factor Surveillance System, and the Massachusetts hospital reduction estimates of Land, et al [10]. Figure 1 is a flowchart that summarizes the stages of this analysis and the data sources used at each stage.

### Patient Population

The patient population is limited to Massachusetts Medicaid beneficiaries aged 18 to 64 years who are smokers. We excluded those enrolled in both Medicaid and Medicare (also known as “dual eligibles”), since most of their inpatient costs are paid by Medicare. The MEPS analytic sample included 805 Medicaid beneficiaries who are smokers. Smokers were defined as those who reported that they are current smokers as of the last year of participation in the survey.

### Analytical Horizon, Perspective, and Setting of the Study

Land's study examined changes in hospital admissions in the period before and after use of tobacco cessation benefits; on average, patients were followed for 70 weeks after they began using tobacco cessation medications [10]. Thus, the time horizon of potential savings is about 1.3 years after the receipt of benefits. Our study does not seek to extrapolate longer term benefits associated with smoking reduction. Nor does it seek to extrapolate to benefits beyond reduced hospitalizations for cardiovascular conditions among Medicaid beneficiaries that smoke. Examples of benefits omitted from this analysis include benefits for other averted diseases, increases in worker productivity, and potential life years saved. It focuses on costs and savings incurred by the Medicaid program in Massachusetts.

### Clinical Benefits and Economic Measures

Our primary clinical benefits are reduced admissions for certain cardiovascular diseases. Land, et al. grouped inpatient admissions into groups that had been defined by the Healthcare Utilization Project (HCUP) using clinical classification software (CCS) codes of 100 for acute myocardial infarction (AMI), 101 for coronary atherosclerosis and other heart disease, and 102 for non-specific chest pain. The same system is used in the MEPS data that we analyzed. These group codes are based on numerous specific CPT-9-CM procedure codes reported in hospital claims records and grouped by the CCS system [20]. It should be noted that non-specific chest pain may have multiple etiologies, which may include cardiovascular problems but might also include other problems, such as reflux disease or pleuritis. Following the CCS and Land, et al., we classified these as cardiovascular problems, but recognize that some could have other etiologies.

Our economic benefit data include costs to the Medicaid program for prescription drugs and counseling costs and savings due to averted inpatient admissions. All costs and savings were converted to 2010 dollars using medical price inflation data from the Bureau of Labor Statistics.

### Data Sources

A variety of data sources were used. Administrative data on program costs were used to compute the annual average cost per patient in implementing the program. Data on program costs for fiscal years 2007, 2008, and 2009 were provided by the Massachusetts Tobacco Cessation & Prevention Program, based on Medicaid (known as MassHealth in Massachusetts) administrative cost data. These included the cost of pharmacotherapy, counseling, and program outreach and promotion for fiscal years 2007, 2008, and 2009.

To compute the economic value of program benefits such as averted hospital inpatient admissions we used data from the *Medical Expenditure Panel Survey (MEPS). To increase the sample size of the study we pooled data from the 2002–8 MEPS. MEPS is a nationally representative survey of non-institutionalized individuals conducted by the Agency for Healthcare Research and Quality. It is a widely used survey* that collects information on socio-demographic characteristics, health services use, health conditions, access to care, health insurance coverage, medical expenditures, sources of payment, and income for each person surveyed, drawn both from surveys of individuals and health care providers. *We restricted t*he analytic sample to unique individuals reported as 18 to 64 year old Medicaid beneficiaries who were current smokers. The MEPS longitudinal design allows repeated observations on the same individuals several times during the year. By restricting the sample to unique individuals we were able to compute robust *standard errors*. The MEPS data reflect a national sample of Medicaid smokers and is one of the few data sets that contain expenditures. (It is worth noting that we could not obtain hospital savings from administrative data; a substantial share of the hospital data from Massachusetts was from managed care systems and lacked cost or expenditure data.)

To adjust the results of the models to reflect the characteristics of adult Medicaid beneficiaries and smokers living in Massachusetts, we used data from the Massachusetts Department of Health's Behavioral Risk Factor Surveillance Survey (BRFSS) for 2007–9. The BRFSS does not contain data on medical expenditures. The state BRFSS survey includes some questions not included in other states' BRFSS data that permits identification of Medicaid smokers. We also used the Consumer Price Index for inpatient hospital data from the Bureau of Labor and Statistics (BLS) to inflate program costs and economic value of program benefits to 2010 dollars.

### Analytical Approach and Models

Figure 1 summarizes the overall flow of analyses in this paper. For the first stage, *we estimated expenditure models for inpatient hospital expenditures for cardiovascular conditions for adult Medicaid beneficiaries who are smokers, using MEPS data. To specify the model, w*e used a modified version of Aday and Andersen's behavioral model of factors affecting health utilization [21]. This model hypothesizes that *utilization* depends on predisposing, enabling and health need factors. The predisposing factors included age, race/ethnicity, gender and marital status. The enabling factors included income as a percent of poverty, educational attainment and health insurance status. Health need factors included self-reported health status (fair or poor health), whether the respondent exercised and obesity status. We also included geographic factors that may affect use of care, including rural/urban status and Census region.

To test the robustness of the models, we tested different specifications. We estimated a version including having a diagnosis of diabetes as an additional health factor and a version with diabetes and hypertension. These variables were not significant in any of the models, so we reverted to our base models.

There are two well-recognized econometric problems in estimating medical expenditures. The first is that there are many zero observations leading to systematic differences in characteristics between patients with zero expenditure compared to those with positive expenditures. The second problem is that medical expenditures are highly skewed because a subset of patients with positive expenditures has very large expenditures [22]–[23]. Two-part models that take into consideration patients with zero expenditures and patient with positive expenditures are typically used to address the problem of many zero observations. However, in our case, we only look at those who have inpatient admissions and virtually all have non-zero expenditures. Hence, there is no need to use the first part of the two-part model, usually logistic or probit regressions, to account for the probability of using any medical care.

To address the skewness in expenditures, we used log-transformed generalized linear models (GLM) with log link and Gamma distribution to estimate direct hospital inpatient expenditures associated with cardiovascular services noted above by adult Medicaid beneficiaries who are also smokers. The log link was incorporated into the model specifically to address the skewness observed in the expenditures data. We developed several models to predict total healthcare expenditures and conducted sensitivity analyses for robustness. We used the diagnostic and specification tests recommended by Manning and Mullahy to select the final models [24]. Final models were adjusted for MEPS' complex survey design and weighting, using the survey design adjustment procedures in Stata 11.

The expenditure models using MEPS data reflect characteristics of Medicaid smokers nationwide. In order to calibrate our estimates to more closely correspond to Massachusetts residents, we then used data from the Massachusetts BRFSS to identify characteristics of adult Medicaid beneficiaries in Massachusetts. We then adjusted our expenditure estimates based on the demographic, socioeconomic, access, behavioral, health status and health condition variables of Massachusetts Medicaid smokers (see Table 1).

**Table 1:** Descriptive Characteristics of 18–64 Year Old Medicaid Beneficiaries Who are Current Smokers.

Variables	U.S.(from MEPS)	Massachusetts (from BRFSS)
**Percent Admitted to Hospital by Diagnosis Group**		
Acute myocardial infarction	1%	3%
Coronary atherosclerosis & other heart disease	1%	2%
Non-specific chest pain	3%	3%
**Demographic Variables**		
**Mean Age**	37.4 years	34.5 years
**Gender**		
Male	29%	42%
Female	71%	57%
**Race/Ethnicity**		
White	69%	66%
Hispanic	10%	17%
Black or African American	20%	9%
Asian	1%	1%
**Marital status**		
Married	27%	33%
Divorced	23%	15%
Widowed	3%	2%
Separated	6%	4%
Never married	47%	44%
**Socioeconomic Status**		
**Income as % of Poverty**		
0–100% of poverty	61%	63%
100–200% of poverty	23%	22%
200–400% of poverty	12%	9%
Over 400% of poverty	0.04	0.06
**Education**		
Less than high school degree	44%	24%
High school graduate	53%	66%
College graduate or more	03%	10%
**Behavioral Variables**		
No physical activity	59%	32%
Physical Activity	41%	68%
Normal weight	41%	39%
Overweight	24%	35%
Obese	35%	23%
**Health Status**		
Excellent/Very good/Good	54%	72%
Fair/Poor	46%	30%
**Morbidity**		
No diabetes	85%	94%
Diabetes	15%	6%
No Hypertension	69%	80%
Hypertension	31%	20%
**Residence/Region**		
Non-Metropolitan Statistical Area	22%	
Metropolitan Statistical Area	78%	

After that stage, we computed cost savings associated with inpatient expenditures related reductions in AMI, acute coronary heart disease, and non-specific chest pain among Medicaid smokers. Costs were based on administrative data provided by Massachusetts officials. All program costs and estimated savings were inflated to 2010 dollars using the Consumer Price Index for inpatient hospital costs from the Bureau of Labor Statistics.

We computed the return on investment (ROI) as:
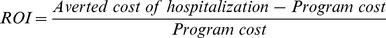
That is, any ROI greater than zero means that more was saved (or gained) than was spent on the initiative.

To assess the uncertainty of the estimates, we computed different levels of ROI by using the 95% confidence intervals of the predicted expenditures for the noted cardiovascular conditions by adult Medicaid smokers into account. This enabled us to compute low, medium and high estimates of the potential savings due to reduced cardiovascular admissions.

## Results

### Descriptive Statistics

In our initial analyses of the 2002–8 MEPS data, 98% of adult Medicaid smokers 18 to 64 who had inpatient hospital admissions also had hospital expenditures reported. (We believe that the 2% without expenditures are due to the fact that MEPS does not report expenditures in cases where certain hospitals provide care without charge, on a “charity” basis.) The average expenditure for a Medicaid smoker's admission was $13,950. However, the average adult hospital in-patient in the U.S. spent about $28,691 with AMI diagnoses, $9,828 for coronary atherosclerosis and other heart disease, and $6,874 for non-specific chest pain.

Table 1 compares the characteristics of the overall sample of adult Medicaid beneficiaries who were smokers at the national level (based on MEPS data) and in Massachusetts (based on BRFSS data), regardless of whether they had an inpatient admission. A slightly higher proportion of Medicaid beneficiaries residing in Massachusetts were admitted for hospital inpatient services for AMI and coronary atherosclerosis and other heart disease, compared to the national average. But these differences were small and not significant. Other socio-demographic characteristics of Massachusetts Medicaid beneficiaries were similar to the national average, except that there were a higher proportion of males among Medicaid smokers compared to the national average. A higher proportion of Massachusetts residents had higher incomes or were college graduates, compared to adults at the national level, probably because Massachusetts has more generous Medicaid eligibility than most other states. In terms of behavioral factors, Massachusetts residents exercised more and reported a lower percentage of adults with obesity compared to the U.S. (though the lower percentage of adults with obesity was offset by higher rates over overweight). Similarly, those in the Massachusetts Medicaid program were more likely to report that they were in excellent, very or good health, and less likely to report diabetes and hypertension than those at the national level.

### Program Costs

As indicated in Table 2, $20,178,210 was spent for medications or counseling under the state's Tobacco Cessation and Prevention Program from FY 2007 to 2009, representing an average of $6,726,070 per year. Additionally, $558,500 was spent on program's promotion and outreach during the three years, representing an average of $186,167 annually. A total of 550,067 individuals who were between 18 and 64 years old participated in the state's Medicaid program during fiscal years 2007–2009, of which 188,123 (34.2%) were identified as smokers. Over 75,000 unique Medicaid beneficiaries participated in the tobacco cessation program during the three-year period. During 2007–9, an annual average of 37,762 participants who were smokers used medications or counseling services. The annual average cost per user of medication and counseling services was $178; an additional $5 was spent on program outreach and promotion. In sum, a total of $183 was spent annually per user to implement the program from 2007–2009.

**Table 2:** Program Costs for Adult Medicaid Smokers Who Participated in the Tobacco Cessation Program during Fiscal Years 2007–2009 (US $ 2010).

Category of Services	Total Program Costs	Annual Average Total Costs	Annual Average Number of Users	Annual Average Cost per User
Medications & counseling	$20,178,210	$6,726,070	37,762	$178
Program outreach and promotion	$558,500	$186,167	---	$5
Total	$20,736,710	$6,912,237	37,762	$183

Source: Based on authors' calculations using data from MassHealth, Office of Clinical Affairs.

### Economic Value of Hospital Inpatient Admissions for Cardiovascular Conditions

As shown in Table 3, results from expenditure models that were calibrated using characteristics of Medicaid smokers in Massachusetts showed adjusted inpatient expenditures of $26,044 for AMI (95% confidence interval from $25,026 to $27,060), of $12,760 for coronary atherosclerosis and other heart disease (95% confidence interval from $12,260 to $13,258) and $7,367 for non-specific chest pain (95% confidence interval from $7,086 to $7,647). The models were adjusted for socio-demographic, socio-economic, access, behavioral, health status and health condition variables of Massachusetts Medicaid smokers, as described in the methods section.

**Table 3:** Estimated (Adjusted) Annual Average Expenditures Per Inpatient for Cardiovascular Conditions for Adult Medicaid Smokers in Massachusetts (US $ 2010).

Cardiovascular conditions	Low	Midpoint	High
Acute myocardial infarction	$25,026	$26,044	$27,060
Coronary atherosclerosis	$12,260	$12,760	$13,258
Non-specific chest pain	$7,086	$7,367	$7,647

To compute the economic value of averted hospital inpatient admissions for cardiovascular conditions by adult Medicaid smokers in Massachusetts (or the benefits of the program), we multiplied the adjusted inpatient expenditures of the each of the conditions by their corresponding rate of reductions in hospital inpatient admissions estimated by Land et al [10]: AMI (46%), coronary atherosclerosis and other related conditions (49%) and non-specific chest pains (32%). Subsequently, we multiplied each of the respective results by the rate of hospital inpatient admissions among Medicaid smokers in Massachusetts, as reported in BRFSS (3% for AMI, 2% for coronary atherosclerosis, 3% for non-specific chest pain). As indicated in Table 4, we found that the economic value of averted hospital inpatient admissions for cardiovascular conditions per adult Medicaid smoker in Massachusetts ranged from $368 to $398 for AMI, from $113 to $117 for coronary atherosclerosis and other heart disease, and from $68 to $78 for non-specific chest pain. This resulted in total program benefits per adult Medicaid smokers in Massachusetts user of $571, ranging from $549 to $593.

**Table 4:** Estimated Annual Value of Averted Hospital Inpatient Admissions for Cardiovascular Conditions Per User in Massachusetts (US $ 2010).

Cardiovascular Conditions	Low	Midpoint	High
Acute myocardial infarction	$368	$383	$398
Coronary atherosclerosis	$113	$117	$122
Non-specific chest pain	$68	$71	$68
**Total**	**$549**	**$571**	**$593**

### Net Savings and Return on Investment

As reported in Table 5, we estimated net annual savings of $388 (ranging from $366 to $410) per user in Massachusetts, compared to program costs of $183 per user. This leads to an annual average ROI per adult Medicaid smoker in Massachusetts of $2.12, with a range from $2.00 to about $2.25. In other words, each $1 spent on medications and counseling, and promotion and outreach for Medicaid smokers was associated with a reduction of $3.12 (range $3.00 to $3.25) in Medicaid expenditures for cardiovascular hospital admissions, resulting in net savings between $2.00 and $2.25.

**Table 5:** Estimated Net Annual Savings Per User and Estimated Return on Investment Associated with Reduced Cardiovascular Admissions among Medicaid Smokers in Massachusetts (US $ 2010).

	Low	Midpoint	High
Net annual savings	$366	$388	$410
**Return on investment**	**$2.00**	**$2.12**	**$2.25**

As noted earlier in this paper, it is possible that some of the admissions due to non-specific chest pain are not actually due to cardiovascular conditions, but disorders like reflux disease or pleuritis. Even if we net out these savings related to non-specific chest pain, the estimated ROI remains highly positive, ranging from $1.63 to $1.84.

## Discussion

The current study advances the literature on the economic evaluation of smoking cessation programs at the state level in the United States. Findings from this study indicate that a well-promoted program of comprehensive access to tobacco medications and counseling implemented in Massachusetts was cost beneficial. Over an average of 70 weeks after beginning to use smoking cessation medications, Medicaid beneficiaries experienced fewer hospital admissions due to cardiovascular conditions, leading to a net annual savings of $366 to $410 per Medicaid user or an ROI of $2.00 to $2.25 during the period of 2007–2009. These results were adjusted for an extensive set of control variables and the findings were robust to different model specifications.

This study has strengths and limitations. In terms of strengths, the study used detailed administrative data about program costs and relied on estimates of reductions in hospital admissions based on detailed hospital data analyzed by Land, et al [10]. Because we lacked actual administrative data on the costs of hospitalizations averted, we used a comprehensive national data set (MEPS) to estimate the costs of cardiovascular hospital admissions among adult Medicaid smokers. To control for variations in the factors associated with expenditures, we controlled for an extensive set of demographic and health characteristics and then calibrated these to correspond the risk profile of Medicaid smokers in Massachusetts, using the BRFSS data. Our study is also limited by the limitations of Land's study [10] which generated estimates of reductions in hospitalization among Medicaid beneficiaries. That paper discussed its limitations, notably the use of claims data as a proxy for health events and of the receipt of the tobacco cessation benefit as a proxy for actual smoking cessation.

A key limitation of our analysis is that we assume that actual hospital savings are equivalent to the average costs per admission multiplied by the number of averted hospital admissions. This may introduce error in two ways. First, it is possible that averted admissions occur among either healthier or sicker patients who have lower (or higher) inpatient expenditures. If, for example, admissions were only averted among healthier patients, more expensive patients would still be admitted and our estimates would overstate cost savings. The second source of error is that in addition to reducing admissions, tobacco cessation programs may reduce the severity of problems among those admitted. In this case, there would be additional savings through the result of reduces expenditures even among those who were hospitalized, which our study has not captured. Our inclusion of a range of hospital expenditures, based on the confidence intervals incorporates some of the uncertainty about the actual savings and the heterogeneity of patient health.

Results from this study are consistent with previous research which has indicated the efficacy and cost-effectiveness of certain drug therapies in reducing smoking and the health benefits of smoking cessation. In particular, it has focused on reductions in medical expenditures related to hospitalizations for cardiovascular disease. It did not measure the long-term or lifetime impacts on medical expenditures. On the other hand, prior analyses have suggested that smoking cessation may be the most cost-beneficial long-term strategy for the reduction of the burden of cardiovascular disease in the United States [25].

### Conclusions and Policy Recommendations

A disproportionate number of smokers in the United States are low-income and insured by Medicaid. Findings from Land, et al. [9]–[10] and from this study suggest that comprehensive tobacco cessation efforts can reduce the prevalence of smoking in a high risk population and reduce net costs for the Medicaid program. This analysis focused solely on medical care savings resulting from reduced cardiovascular admissions among program participants. For example, it did not estimate potential health improvements or savings that might be associated with reduced second hand smoke exposure for family members or intrauterine exposure from pregnant smokers. Nor did it consider other potential savings, such as the reduced burden to low-income families from the cost of purchasing cigarettes or the potential for improved productivity and confidence associated with quitting smoking.

It is well understood that it is difficult to stop smoking and that while many may successfully quit in the short- term, there is a substantial risk of recidivism. While we cannot be assured that Medicaid beneficiaries who quit smoking remain abstinent in the long run, there appear to be near-term reductions in smoking rates that lead to near-term Medicaid savings within the following year or so. These are conservative estimates given that we only measured short-term benefits associated with reductions in inpatient hospital admissions due to cardiovascular conditions. But program administrators are often most interested in near-term savings, since they do not know how long beneficiaries will remain covered by Medicaid and because fiscal concerns lead to pressure for near-term savings.

Both the federal and state governments share in the costs and savings related to stronger tobacco cessation efforts for Medicaid beneficiaries. Although both the federal and state governments are under substantial budgetary pressure, this research suggests that further investments in comprehensive tobacco cessation under Medicaid would be a sound investment that reduces medical expenditures relatively quickly. As noted earlier, the Patient Protection and Affordable Care Act already includes efforts to strengthen tobacco cessation services in Medicaid, including mandatory coverage of comprehensive services for pregnant women and enhanced coverage of pharmacotherapy for smoking cessation. Moreover, Medicaid coverage is scheduled to expand to serve millions of additional low-income non-elderly adults in 2014 [26]. Thus, tobacco cessation services in Medicaid could soon be offered to a much larger share of the low-income smoking population.

Despite the budgetary problems faced by Medicaid program administrators and state and federal officials, efforts to implement comprehensive tobacco cessation programs for Medicaid enrollees (not just those who are pregnant) may be an element of evidence-based policy to both improve public health and reduce health care expenditures. Because Medicaid provides health insurance coverage, including coverage for preventive services, for a very large share of a high-risk, low-income population, public health objectives include recommendations for comprehensive smoking cessation coverage under Medicaid [4]. Research concerning the efficacy and cost-effectiveness of these initiatives to encourage smoking cessation may provide valuable information to policymakers and researchers alike. Additionally, cost-effectiveness studies that account for heterogeneity in populations of smokers are needed to provide important information to policymakers and other key stakeholders.
